# Bonding Effectiveness of Saliva-Contaminated Monolithic Zirconia Ceramics Using Different Decontamination Protocols

**DOI:** 10.1155/2024/6670159

**Published:** 2024-04-04

**Authors:** Necla Demir, Ozge Genc, Ipek Balevi Akkese, Meral Arslan Malkoc, Mutlu Ozcan

**Affiliations:** ^1^Selcuk University, Faculty of Dentistry, Department of Prosthodontics, Konya, Türkiye; ^2^Akdeniz University, Faculty of Dentistry, Department of Prosthodontics, Antalya, Türkiye; ^3^Clinic for Fixed and Removable Prosthodontics and Dental Materials Science, Center for Dental and Oral Medicine, University of Zurich, Zurich, Switzerland

## Abstract

**Objective:**

This research study investigated the effect of new decontamination protocols on the bonding capacity of saliva-contaminated monolithic zirconia (MZ) ceramics cemented with two different monomer-containing self-adhesive resin cements.

**Materials and Methods:**

Standardized tooth preparations (4 mm. axial height) were performed for eighty human maxillary premolars under constant water cooling system. Eighty monolithic zirconia crowns (Whitepeaks Supreme Monolith) (*n* = 8/10 groups) were manufactured by CAD-CAM. Specimens were kept in the artificial saliva at pH = 7.3 for 1 minute at 37°C except control groups. The specimens have not been prealumina blasted and grouped according to cleaning methods and resin cements: control groups (C) (no saliva contamination + GPDM + 4‐META (N) (CN) and 10-MDP (M) containing resin cement (CM), alumina blasted (AL) + GPDM + 4‐META (ALN) and 10-MDP containing resin cement (ALM), zirconium oxide containing universal cleaning agent (IC) applied + GPDM + 4‐META (N) (ICN) and 10-MDP containing resin cement (ICM), pumice (P) applied + GPDM + 4‐META (PN) and 10-MDP containing resin cement (PM), and air-water spray (AW) applied + GPDM + 4‐META (AWN) and 10-MDP containing resin cement (AWM)). Monobond Plus was applied to all surfaces for 40 seconds before cementation. The thermal cycle was applied at 5,000 cycles after cementation. The crowns were tested in tensile mode at a speed of 1 mm/min. The mode of failure was recorded. SEM examinations were carried out at different magnifications. Data were analyzed using rank-based Kruskal-Wallis and Mann–Whitney tests.

**Results:**

No significant differences were found between the surface treatments and between the two types of resin cements. Interaction effects between surface treatments and resin cements were found to be significant by two-way ANOVA analysis. ICM group resulted in significantly better bond strength results compared with CN. ICM was found to result in better bond strength results compared with PM. The combination of universal cleaning agent and 10-MDP containing resin cement had significantly the highest cementation bond strength values. The increasing order of mean tensile bond strength values of decontamination protocols was C < AW < P < AL < IC. The mean tensile bond strength of 10-MDP containing resin cement was slightly higher than GPDM + 4‐META containing resin cement.

**Conclusions:**

Universal cleaning agents can be preferred as an efficient cleaning method with 10-MDP-containing cement after saliva contamination for better adhesive bond strength of 4 mm crown preparation height of monolithic zirconia ceramics.

## 1. Introduction

Recently, the use of monolayered monolithic zirconia ceramics (MZ) in prosthetic dentistry has become very popular as a consequence of its favourable biocompatibility and aesthetics/mechanical properties [1]. Despite their wide applicability in restorative and prosthetic dentistry, the clinical success of zirconia restorations is affected by the durability of the bond strength between zirconia and the tooth structure [2]. Meanwhile, another major problem about the retention of ceramic crowns can be explained with the possibility of saliva contamination before cementation. To maintain reliable bond strength, bonding procedures must be done in saliva-contaminant-free surfaces. Zirconia has a high affinity for phosphate, which is found in saliva [3]. During clinical adjustments of ceramic crowns, contamination of the intaglio of ceramic restorations by saliva, blood, or silicon indicators cannot be prevented [3, 4]. Alumina-blasting (AL) of the intaglio of zirconia restorations with Al_2_O_3_ nanoparticles is evaluated to be the only efficient cleaning method that allows proper adhesive bonding after saliva contamination [3]. Air abrasion improves surface energy and wettability, required as a prior condition that strengthens the adhesive bonding [5]. Therefore, we aimed to compare the effectiveness of alumina-blasting, a recently developed universal cleaning agent (Ivoclean), and also pumice application as a different decontamination protocol on bond strength for monolithic zirconia (MZ) crowns.

Saliva consists of phospholipids which consists of phosphate groups, which highly adhere to the surface of prosthetic restorations. The universal cleaning agent (IC) (Ivoclar Vivadent, AG) contains high levels of zirconia in a NaOH solution. It eliminates the saliva particles from the intaglio surfaces of zirconia which can strengthen adhesion of the resin to MZ [6]. The supplied products' directions suggest to apply air/water spray to efficiently eliminate saliva from a surface including MZ crowns. Initial assessment of this product has demonstrated widespread success [7]. Its technology uses a basic solution of MZ oxide (Ivoclar Vivadent Scientific Documentation, 2011) which tends to adhere to phosphate and subsequently decontaminates surfaces. However, in the researchers' mind, clinicians usually overdo decontamination procedures to maintain a strong bonding between resin cement and dental crowns [7]. Thus, the aim of this study was to determine the bonding performance of saliva-contaminated ceramics by applying alumina-blasting and the previously mentioned cleaning agents. The cleaning performance of such cleaning methods compared to alumina-blasting on resin cement adhesion to saliva-contaminated MZ ceramics is not known enough. Consistent adhesion of zirconia ceramics was acquired through mechanical and chemical retention of resin to the substrate [8]. Mechanical retention was maintained by enhancing the adhesive surface with alumina-blasting (AB). Chemical and long-term durable cementation to MZ ceramic was approved for phosphate monomer ceramic primer [9] or specifically 10-methacryloyloxy-decyl-dihydrogenphosphate (MDP). However, the durability of 4-META or GPDM containing dual-cure self-adhesive resin cements is not enough investigated. Supporting this idea, after treating the intaglio surface of MZ crowns with MDP-containing primer Monobond Plus, we planned to compare the self-adhesive resin cement which contains 4-META or GPDM monomer with the other 10-MDP containing self-adhesive resin cement.

There is a lack of investigation concerning the cleaning ability of surface treatments and cleaning agents especially IC used on the zirconia surfaces without prealumina-blasting, and also, there is no research study related with the cleaning capability of pumice application on monolithic zirconia surface. The novelty of the study is to reveal the cleansing effect on saliva-contaminated monolithic zirconia surface by using only IC agent and pumice application.

The goal of this report was to investigate the effect of the universal decontamination cleaning agent, AB, air-water/spray, and pumice application on tensile adhesion strength of two different self-adhesive dual-cure resin cement materials to MZ ceramic exposed to artificial saliva. The null hypothesis tested was that there is no bonding effectiveness difference between the new cleaning agent, AB, air/water spray, and pumice application and the two different monomer-containing self-adhesive resin cements used for monolithic zirconia ceramic crowns' height of 4 mm.

## 2. Materials and Methods

### 2.1. Specimens' Preparation

According to the results of the power analysis (G∗Power software v3.1.10), a requirement of at least 8 specimens in each group was determined with 95% confidence (1-*α*), 80% test power (1-*β*), and *f* = 0.4 effect size. For this reason, 8 samples were prepared for each subgroup, and 5 surface treatment groups were divided into two subgroups according to the resin cement treatment. Eighty human maxillary premolars were used in the study, and caries and/or prior repairs were not included. Teeth with comparable dimensions (±1 mm) including buccolingual, mesiodistal, and coronoapical were included. Teeth were kept in a 37°C incubator to simulate the oral environment until mechanical tests were initiated. The teeth were individually embedded in acrylic resin (Imicryl, Turkey). The tooth was embedded in a cylinder made of stainless steel perpendicular to the block base, while exposing the root. Teeth were prepared with a high-speed diamond rotary cutting bur, while cooling. The overall technical dimensions were a 4 mm crown length and 1 mm peripheral rounded shoulder at a low taper angle of approximately 6° and positioned 0.5 mm over the cementoenamel junction. The preparation was located partially to dentin. Each tooth was scanned using an optical digital scanner (Dental Wings Inc, Montreal, Canada). The shape of each tooth was digitized three-dimensionally (Whitepeaks Supreme Monolith, Germany) (*n* = 8) for CAD-CAM fabrication (DWOS, Dental Wings Inc, Montreal, Canada) of 80 monolithic zirconia crowns, and horizontal ring structures were designed for tensile bond strength test. Artificial saliva (AS) was prepared by mixing 2000 mg/L C_8_H_8_O_3_, 10000 mg/L Na CMC (C_8_H_15_NaO_8_), 58.87 mg/L MgC_l2_.6H_2_O, 166.11 mg/L CaCl_2_.2H_2_O, 417.6 mg/L K_2_HPO_4_, 624.31 mg/L KCl, and 0.05 mg/L F as Yoshida. All materials used are summarized in Table 1. Except for the control group, all samples were immersed in artificial saliva at pH = 7.3 for one minute at 37°C and rinsed with water followed by air drying, both for 15 seconds. The samples were randomly divided into 5 groups and 2 subgroups for each group using a centralized, computer-generated randomization system. 10 groups (*n* = 8) were assigned as control groups (C) (no saliva contamination + GPDM + 4‐META (N) (CN) and 10-MDP (M) containing resin cement (CM), alumina blasted (AL) + GPDM + 4‐META (ALN) and 10-MDP containing resin cement (ALM), zirconium oxide containing universal cleaning agent (IC) applied + GPDM + 4‐META (N) (ICN) and 10-MDP containing resin cement (ICM), pumice (P) applied + GPDM + 4‐META (PN) and 10-MDP containing resin cement (PM), and air-water spray (AW) applied + GPDM + 4‐META (AWN) and 10-MDP containing resin cement (AWM)).

**Figure 1:**
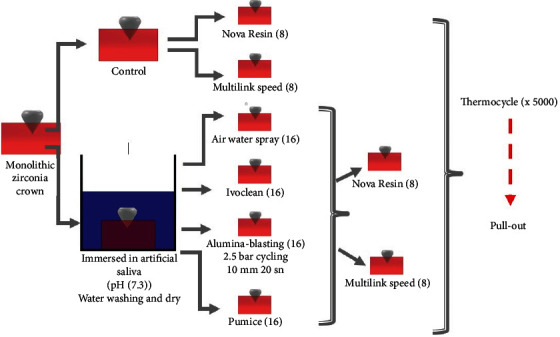
Schematic illustration of the method of the study.

The contamination and cleaning procedures are summarized in Figure 1. After these treatment methods, a primer consisting of silane and phosphate monomers (Monobond Plus; Ivoclar Vivadent, Schaan, Liechtenstein) was applied to all conditioned surfaces for 40 seconds. Finally, GPDM + 4‐META containing (Nova Resin) and 10-MDP containing (Multilink Speed) dual-cure self-adhesive resin cements were used for luting procedure. The crowns were cemented on the abutment teeth using finger pressure. Excess resin was removed by micro brush followed by light curing of each surface of the crowns for 40 seconds with a LED curing unit (Elipar S10, 3M ESPE, Germany). A coating of glycerin was applied to each crown margin to reduce oxidation. The embedded teeth were subjected to continuous water spray to keep the teeth cool during the resin polymerization.

### 2.2. Specimen Aging

All specimens were then kept at 37°C for 24 hours in a water bath and subjected to 5000 thermal cycles in distilled water bath between 5°C-55°C at 30 seconds and 5-second transfer time between baths.

### 2.3. Fracture Strength Measurement

**Figure 2:**
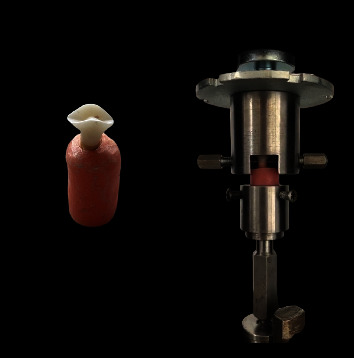
The MZ crown specimen and the tensile bond strength test.

The horizontal ring of the crown supported by a designed stainless steel holder was used to remove the crowns from the insertion axis. The tensile bond strength was measured with a testing machine (DVT Devotrans GP, Turkey) at a crosshead speed of 1 mm/min (Figure 2). The dislodgement values recorded in Newton (N) were converted to the tensile bond strength in MPa unit by dividing the surface area (mm^2^) of the prepared tooth which was calculated using the following formula [10]:

(1) $$\begin{array}{c} \text{Area}=\frac{\pi }{4d1^{2}}+\pi \frac{h}{2}\left(d1+d2\right)+\frac{\pi }{4}\left(d3^{2}-d2^{2}\right), \end{array}$$

where *d*1 is the diameter at the top of the preparation, *d*2 is the diameter at the base of the preparation, *d*3 is the diameter of the base of the preparation plus 1 mm margin on either side, and *h* is the axial height.

### 2.4. Failure Types' Analysis

After the tensile test, the specimens were examined under an optical microscope (SZ-PT Olympus, Japan) at a magnification of ×15 to define the location of failure surface. Failure modes, such as adhesive, cohesive, or mixed fractures, along the high translucency ceramic surface, were determined by percentage of bonded area. Then, the specimens were dehydrated in increasing concentrations of ethanol and water up to 100% ethanol. All specimens were kept in at 37°C distilled water in an incubator (Nuve Incubator EN 120, Turkey) for 24 hours until all residual moisture was removed. Specimens were gold sputter-coated and observed by using scanning electron microscope (SEM) (Carl Zeiss Evo LS10/Germany) at 15 kV of accelerating voltage, and the working distance was kept at 8-12 mm from mantle surfaces of teeth. Scanning electron microscopy examinations were carried out at 80x, 500x, and 1,000x magnifications.

### 2.5. Statistical Analysis

The tensile bond strength (Fmax) test was statistically assessed with IBM SPSS V23 computer software. The normality of Fmax values was determined by the Shapiro-Wilk test. Following the calculation of mean and standard deviation values, the groups were compared using the two-way ANOVA test. Multiple comparisons of the groups were made with the Tukey test (HSD). The significance level was taken as *p* < 0.050.

## 3. Results

### 3.1. Fracture Strength

**Figure 3:**
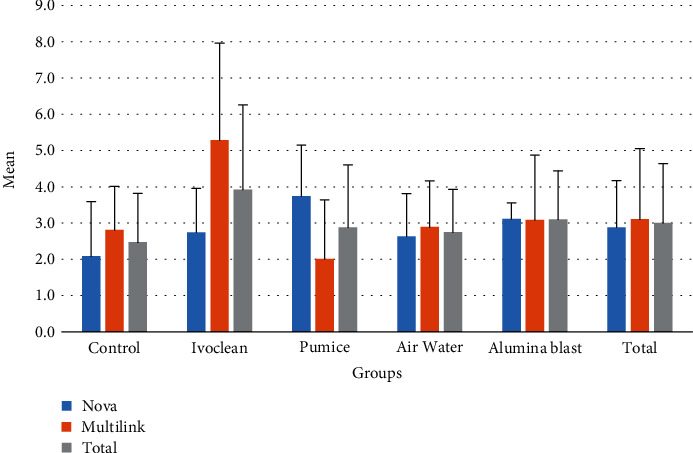
Mean ± SD values of tensile bond strength values of groups.

No significant statistical differences were observed for the cementation agents (*p* = 0.091). The mean bond strength for Nova Resin Cement was lower (2.88 ± 1.29 mPa) than the Multilink Speed (3.11 ± 1.94 mPa) (Table 2). No significant statistical differences were also observed for the cementation agents (*p* = 0.324). The mean bond strength was 2.47 ± 1.35 mPa for controls, 3.92 mPa ± 2.34 for Ivoclean (IC) group, 2.88 ± 1.72 mPa for pumice (P) group, 2.75 ± 1.18 mPa for air-water (AW) group, and 3.1 ± 1.34 mPa for the alumina blast group (AL) (Table 2 and Figure 3). The increasing order of mean tensile bond strength values of decontamination protocols was C < AW < P < AL < IC. Interaction effects between groups and subgroups were found to be significant by two-way ANOVA analysis (*p* < 0.05). ICM group resulted in significantly better bond strength results compared with CN. ICM was found to result in better bond strength results compared with PM. The combination of universal cleaning agent and 10-MDP containing resin cement had, significantly, the highest cementation bond strength values.

### 3.2. Failure Type Analysis

**Figure 4:**
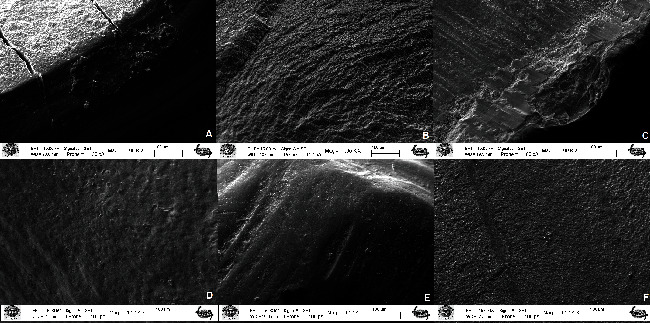
SEM images of the debonded tooth surfaces after surface treatments: (A) air-water spray (Multilink Resin cement-mixed failure mode), (B) Ivoclean (Multilink Resin cement-cohesive failure mode), (C) control (Multilink Resin cement-mixed failure mode), (D) control (Nova Resin cement-adhesive failure mode), (E) alumina-blasting (Multilink Resin cement-adhesive failure mode), and (F) alumina-blasting (Multilink Resin cement-cohesive failure mode).

SEM images of the treated surfaces of the teeth were examined following tensile bond strength test at a magnification of 1,000 (Figure 4). The frequency osf mixed failure mode was mostly observed in all samples of IC, AL, and P groups for the two types of self-adhesive resin cements (Table 3). While adhesive failure was rarely observed in the groups for both types of cements, cohesive failure was observed with more than adhesive failure in all groups after being contaminated with artificial saliva (Table 3).

## 4. Discussion

The goal of this study was to investigate the bond strength of different self-adhesive resins on saliva-contaminated MZ surfaces following surface treatments. The null hypothesis tested that there is no bonding effectiveness difference between the different surface treatments, and the two different monomers containing self-adhesive resin cements used for saliva-contaminated MZ crowns were rejected. The effect of saliva contamination on ceramic adhesion by simulating a clinical try-in procedure was explored. We used artificial saliva at pH 7.3 because obtaining natural saliva is extremely complex due to multiple variables based on the time of day collected. Exact duplication is impossible [11]. It has been indicated in different studies that saliva impurities that remain on the surface cannot be completely removed by air-water spray procedure [3, 12, 13]. The results for retention strength after water cleaning are not congruent with other ceramic studies where water led to lower retention force [3, 14, 15]. We posit that, in our study, the chosen procedure of active water spray resulted in higher kinetic energy on the surface and better minimized saliva contamination compared with control group insignificantly. In this study, the reason why the tensile bond strength in the samples applied air-water spray was higher than the control group may also due to the use of artificial saliva. Also in this study, the samples were not alumina blasted before being contaminated with artificial saliva. In other studies, air abrasion before contamination may have caused greater penetration of saliva contaminants by causing roughness on the surface [4, 7, 13, 16], and this situation potentially challenges the effectiveness of the cleaning methods [17]. Additional particle abrasion could negatively affect the long-term durability [16]. However, the effects of cleaning methods such as universal cleaning agents and pumice are not widely investigated. In this study, pumice, which is generally used for cleaning the tooth and composite surface [18], was used as another cleaning method for the ceramics' intaglio surface innovatively. In Kwak et al.'s study, the authors suggest, prior to orthodontic bracket bonding, a simple surface cleaning of the zirconia glaze layer with a prophy cup and pumice. As a result, pumice did not improve the shear bond strength of the brackets as we found in our study [19]. The use of cleaning agents on the inner surface of the dental ceramics with the help of micro brushes can be considered [7, 13]. Ivoclean is a highly alkaline (pH = 13) solution applied extraorally on zirconia surfaces for cleaning before cementation [20]. Applying universal cleaning agents was found to be an effective method for decontamination of the specimens according to the findings of Kim et al. [7]. Hajjaj and Alzahrani [20] also found in their study that the mean SBS of Ivoclean and air-particle abrasion groups was significantly higher than water rinsing and ZirClean™ groups. Tian et al. also approved that Ivoclean or Katana Cleaner is useful for decontamination of both saliva and blood-contaminated zirconia during the intraoral try-in stage to recover the original bond strength of cementation [21]. Sulaiman et al. [22] also concluded in their study that air-borne particle, zirconia cleaning solutions, and hydrofluoric acid are feasible to decontaminate the zirconia surface from saliva prior to bonding the restoration. Awad et al. [23] also stated that 10-MDP-containing cleaner (Katana Cleaner) and zirconium oxide-containing cleaner (Ivoclean) could eliminate the negative effect of saliva and blood contamination on resin-zirconia SBS. The fact that zirconia restorations have complex surface geometry, air abrasion devices are not available in every clinic, or the air abrasion process is not preferred by clinicians because it pollutes the environment and makes it difficult to perform the air abrasion process during clinical trials. Alternatively, non abrasive cleaning solutions have been developed to decontaminate the bonding surfaces of prosthetic restorations after intraoral try-in [16]. Therefore, applying a cleaning agent to the bond surfaces of the ceramics after saliva contamination may be considered to be a more suitable method of cleaning and strengthening the cementation bond [14]. Martinez et al. [16] also concluded in their study that the cleaning paste application was the most effective method in removing saliva contamination. Previous studies used human saliva, and the samples were alumina blasted before being contaminated with saliva [2, 4, 7, 14]. Therefore, there are no researches showing the influence of universal cleaning agents alone (without prealumina-blasting on MZ) on the removal of saliva contaminants and bond strength. Additional researches are recommended to determine if cleaning agents are better than air abrasion in terms of clinical adhesion strength. Zirconia surfaces are highly hydrophobic and have low surface energy, and AB increases the surface energy and provides micro retention [17]. Nevertheless, AB create surface defects such as flaws, plastic deformation, embedded abrasive alumina, and microcracks, which can compromise the mechanical properties of zirconia and decrease fracture strength. Because zirconia ceramics exhibit stress-induced transformation, air-particle abrasion may transform the surface structure and possibly influence its long-term performance. Therefore, exploring alternative methods and a substitute to alumina air-particle abrasion to promote strength and durability of the resin-zirconia bonding interface without damaging the zirconia surface has become a challenge. However, air-particle abrasion methods alone are not adequate for resin cement adhesion to zirconia ceramics [16, 24]. Ahmed et al. demonstrated that Monobond Plus can enhance the retention of zirconia copings [25]. This silane-primer application is also preferred in this study after treatment methods before resin cementation. As Samran et al. [1] indicated the functional phosphate monomers, MDP in the resin cement established a strong chemical bond to ZrO2 particles in the universal agent (IC) than the functional phosphate monomers incorporated in the other investigated self-adhesive resin cements [1]. The interaction between 10-MDP and zirconia was validated by nuclear magnetic resonance (NMR) to be associated with the formation of ionic bonds and hydrogen bonds [22]. The carbon chain in 10-MDP contains a C=C bond that can polymerize with methacrylate resin in the resin cement. Because of the affinity of phosphate for zirconia, zirconium-containing materials such as mesoporous zirconium oxide (ZrO2) and zirconium phosphate (ZrP) have been used to remove phosphate pollutant from polluted water in the field of water pollution control [22]. A zirconium oxide column has also been used in mass spectrometry for highly selective phosphopeptide enrichment [22]. ZrO2 particles containing universal cleaning agent and 10-MDP containing resin cement combination, in turn, might have also manifested in markedly higher bond strength to zirconia surface in our study. In this research study, thermal cycle [26] was also applied before the tensile adhesion strength test to imitate the clinical impact of the oral environment. Thermo-cycling (5,000 cycles) represents 6 months of clinical prognosis of the restorations [27]. In compliance with Johnson et al., the circumference of the preparation [28–30] was made equal for all samples using anatomically similar premolars. The frictional resistance for tooth and crown should also be considered. Compared to a 10° taper, a taper of ~6° may have caused frictional retention of crown at the circumference of the preparation [31, 32]. The cement space was fixed at 70 *μ*m for all samples according to the default setting of the CAD/CAM software. Thus, the minimum thickness of crown at occlusal surface was expected to be 40.5 *μ*m as a result of the subtraction of cement space from occlusal reduction [33]. The technique performed to measure prepared tooth surface area may have affected the outcomes. Some researchers have correlated the weight of tin foil wrapped around the preparation or scanned the prepared abutments with a Cerec 3D camera, and their adhesion area was estimated with the Cerec 3 volume program [33, 34]. In our study, the adhesion area was determined by formula for a truncated cone whereby the area of the flat occlusal surface was also taken into account as described by Palacios et al. and Karimipour-Saryazdi et al. [10, 35]. This would have affected the tensile adhesion strength, making data comparison with other studies challenging [10, 35]. In the examined SEM images, mixed failure type was mostly observed in all samples, but cohesive and rarely adhesive failures were also observed. These results are consistent with the adhesion strength obtained as in past reports [2, 4]. The mixed failure type seen in the samples shows that the bond strength between the zirconia and the adhesive resin is greater than the bond strength within the adhesive resin itself [36]. The adhesive failure type, which is rarely seen in the study with decreasing bond strength values, shows that the contaminants that are not removed from the zirconia surface can prevent the chemical retention between the adhesive resin and zirconia, or the connection interface can be further destroyed during thermal cycling [37]. There are some limitations of this study. We gave static dislodging force, but these forces in the oral environment are dynamic and also not always vertical [38]. The influence of the applied surface technique and agents on the surface roughness is not examined as another limitation. The quantitative measurement data of the elements detected by EDS or XPS analysis of the specimen surfaces can be evaluated in future studies. As a limitation, the dentinal tubule orientation, the number, size, and degree of intra-tubular mineralization vary between human teeth [39]. On the other hand, Mannocci et al. have suggested that dentinal tubules exert only a minor influence on the mechanical properties of dentin [39]. The results of the study that we have obtained should be also supported by studies using human saliva.

We also planned to compare the self-adhesive resin cement which contains 4-META or GPDM monomer with the other 10-MDP containing self-adhesive resin cement. Calamita et al. aimed to evaluate the functional monomers 10-MDP and GPDM to Y-TZP zirconia in their recent study. Despite that, few studies evaluate the influence of different functional monomers except 10-MDP on the zirconia bonding. The results demonstrated that GPDM provides a suitable resin-composite bonding to zirconia in the three concentrations evaluated, with similar bonding to the MDP primers. The mechanism of adhesion between GPDM and zirconia is similar to 10-MDP with the phosphate group in the GPDM molecule interacting with the Zr-OH of the ceramic forming an initially stable bonding. It can be concluded that 10-MDP and GPDM and combining both monomers can provide similar results to the zirconia bonding to resin materials. Nevertheless, 10-MDP is the most efficient component among the cited ones on the chemical adhesion to the dental substrate as we have found in our study [40].

## 5. Conclusion

If universal cleaning agent is applied after saliva contamination, a strong resin bond can also be obtained like as in alumina-blasting by using primer and resin cement containing 10-MDP. Universal cleaning agents can be preferred as an alternative cleaning application with 10-MDP-containing cement after saliva contamination. Universal cleaning agent can be alternatively applied after saliva contamination, and a strong resin bond can also be obtained like as in alumina-blasting by using primer and resin cement containing 10-MDP. The use of pumice or water for cleaning saliva-contaminated dental zirconia is not sufficient for bond strength compared to universal cleaning agent and alumina-blasting. Universal cleaning agents are useful for decontamination of saliva-contaminated zirconia during the intraoral try-in stage to recover the original bond strength of cementation.

## Figures and Tables

**Table 1:** Materials, manufacturer, batch number, and composition.

Materials		Manufacturer	Composition
MZ	Whitepeaks Supreme Monolith	Whitepeaks Dental Solutions GmbH & Co. KG, Germany	ZrO_2_, FeOH_2_, Y_2_O_3_, Al_2_O_3_, ER_3_O_3_, CO_3_O_4_

Universal primer	Monobond Plus	Ivoclar, Liechtenstein	Silane methacrylate, phosphoric methacrylate, and sulfide methacrylate

Resin	Multilink Speed Dual Cure	Ivoclar, Liechtenstein	10-MDP, 5-NMSA, SiO2, HEMA, barium-treated glass powder, sodium fluoride, benzoyl peroxide, dimethacrylates, ytterbium trifluoride, copolymer, glass, silicon dioxide adhesive monomer, initiators, stabilizers and pigments

Resin cement	Nova Resin Dual Cure	Imicryl, Turkey	Hydrophoblic dimethacrylates (matrix) and acid acrylates, glycerol phosphate dimethacrylate (GPDM), 4-methacryloxyethyl trimellitate anhydride (4-META) The filler (more than 70% w/w) is barium glass, silicon dioxide, fluoro-alumino silicate, catalysts, and stabilizators

Clean paste	Ivoclean	Ivoclar Vivadent, Schaan, Liechtenstein	Zirconium oxide, water, polyethylene glycol, sodium hydroxide, pigments, additives

**Table 2:** Mean ± SD values of tensile bond strength values of groups.

	Multilink	Nova	Total
Air-water-Nova	2.89 ± 1.27^ab^	2.63 ± 1.18^ab^	2.75 ± 1.18
Alumina blast	3.09 ± 1.78^ab^	3.12 ± 0.44^ab^	3.1 ± 1.34
Ivoclean	5.29 ± 2.67^a^	2.74 ± 1.22^ab^	3.92 ± 2.34
Control	2.81 ± 1.2^ab^	2.09 ± 1.5^b^	2.47 ± 1.35
Pumice	2.01 ± 1.63^b^	3.74 ± 1.41^ab^	2.88 ± 1.72
Total	3.11 ± 1.94	2.88 ± 1.29	3 ± 1.64

^a,b^Means with the same letter are not statistically different.

**Table 3:** Failure modes of groups.

	Nova resin			Multilink speed		
	Adhesive	Cohesive	Mix	Adhesive	Cohesive	Mix
Control group	2	1	5	1	2	5
Air-water spray	_	2	6	1	2	5
Pumice	_	2	6	_	1	7
Ivoclean	1	2	5	_	4	4
Alumina-blast	2	4	2	1	2	5

## Data Availability

The statistical data used to analyze the findings of this study are explained within the article. The raw datasets generated or analyzed during the current study are available from the corresponding author on reasonable request. The data used to support the findings of this study are included within the article. The surface treatment and cleaning methods, type of resin cements, and the composition of all materials used in the study are included within the article.

## Additional Points

*Clinical Significance*. The universal cleaning agent can be alternatively applied after saliva contamination, and a strong resin bond can also be obtained like as in alumina-blasting by using primer and resin cement containing 10-MDP.
